# Influence of Christianity on Medical Science

**Published:** 1880-05

**Authors:** John Cumming

**Affiliations:** London


### INFLUENCE OF CHRISTIANITY ON MEDICAL SCIENCE.

(BY REV. JOHN CUMMING OF LONDON.)

**Ever** since Jesus suffered, wrought miracles, healed the sick, tilled the ocean, and showed his control over rebellious nature —by bringing it back again into order,—man has gained by degrees a greater mastery over all things, as if then humanity received a new impulse ; and in proportion to his Christian light (I do not say Christianity is the cause, but it certainly is a coincidence,) has been his civilization ; and, in proportion to that, the gradual authority which he seems to be regaining over that nature, the reins of which he lost in Paradise, but which Jesus has now partially, and will again completely put into his redeemed and sanctified hand. It is to me a most delightful experience, to see any one discovery in science or in art, which restores to man, however slightly, the mastery over created things. Is it not true that since Jesus healed the sick, there has been given a greater impulse to curative science than ever was felt before ? Is not medicine, with all its defects, with all the obloquy cast upon it, because it cannot do everything, progressive ? Is it not true, that some diseases, once thought incurable, are now almost extirpated ? Small-pox is now, not only curable, but almost banished from our land. And was the discovery of this mode of cure simply chance ? Will you say it was accident ? I believe it to have been as much an inspiration of the God of providence as the bible is an inspiration of the God of grace. Is it not fact, that man's life is longer than it was ? If you do not believe me, ask the Insurance Societies, and they will tell you it is so by some six years. It is much longer than this, if we remember, that the sickly and delicate infant which was lost before, while only the strong ones survived, is now spared, and, under the blessing of God, and by the appliance of art, grows up to manhood. Is not all this gain ? Is it not progress in the direction in which the miracles of Jesus lay, and in the reversal of that curse which "brought death into the world, and all our woe ?" Is it not also true, that operations once thought perfectly impossible, are now performed by our surgeons with safety and success ?

